# Resolutions of American Academy of Dental Science

**Published:** 1897-07

**Authors:** 


### Resolutions of American Academy of Dental Science.

The Academy, viewing with dismay the character of the advertisements appearing in some of the self-styled Dental Journals, whereby secret preparations, often of a highly dangerous character, are paraded in such company and guise as to deceive those not accustomed to scrutinize closely all medicines thus offered; and, more particularly, of advertisement soliciting dentists to advertise, announcing that "Professional dignity and good advertising will work well together," giving the name and address of the professional "Writer of Dentists' Advertisements," and the unscrupulous acceptance of the above mentioned journals of advertisements, the character of which is detrimental in the highest degree to the advancement of our profession, the best element of which is striving with self-sacrificing and untiring labor to make it worthy the name and title of a Liberal and Learned Profession, therefore,

*Resolved:* That the Fellows of the American Academy of Dental Science strongly condemn such advertising, believing that it is degrading and injurious to the good name of the honorable calling they represent; and they further declare that the editors of such journals, allowing the common tricks of trade to dominate that which should be governed by professional dignity, are unworthy to be acknowledged as teachers and respected confreres among dentists.

*Resolved:* That this Resolution be forwarded to the editors of the leading Dental Journals, as expressing the sentiment of the Academy.

